# Talking Health and Acting Health

**Published:** 1923-09

**Authors:** 


THE
### HOSPITAL and HEALTH
REVIEW

*Established 1886* *No.* 1866. *Vol. LXXII.*

*New Series.* Vol. II No. 24 *September,* 1923. Price Sixpence.

CONTENTS.

### TALKING HEALTH AND ACTING HEALTH.

If we except what Sir Walter Scott would have called the "big bow-wow" subjects of international politics, it is safe to say that there is no topic that is more constantly discussed than the public health. Especially at this season there is a continuous succession of Congresses and Conferences in which everything immediately or indirectly concerning the public health, from dustbins to diabetes, is debated and illustrated. If talk would make us a robust and disease-free people, the prospect would be rosy indeed. A plethora of talk is, no doubt, a necessary evil, and it is only by saying the same thing very often and very loudly that we at last succeed in getting something done, whatever the desired direction. Next month the country will be asked, as it has been asked annually since 1912, for a whole week to talk and write about health. The object of "Health Week" is to force this great subject upon public attention, to convince the individual that, in his degree, he is as closely responsible as Mr. Neville Chamberlain himself for the physical well-being of his fellows. Health, like charity, begins at home, in the sense that unless we seek health and ensue it in and for ourselves, we are unlikely to realise its primary importance to the community. We need to create an impulsive force which will carry us along to the desired haven, the temple in which the goddess Hygeia sits enthroned in a splendour all too solitary. It would, however, be worse than useless to write and talk, however earnestly, about sanitation and the prevention of disease during one week in the year and then relapse into quiescence. Steady, continuous work at arousing the public conscience is alone likely to make it clear to everybody that in this matter we are emphatically our brother's keeper, and "Health Week" is meant, by a brief intensive period, to show how this can be done all the year round.

One of the first lessons to be learned in this connection is how to counteract the unhappy consequences of our having become an urban people, and having long been content to allow our lungs to be clogged with smoke, fog, and all manner of impurities. "Modern civilisation," said the President of the British Medical Association, recently, "is suffocating itself in the polluted atmosphere and darkness of our great industrial cities." Mr. Childe reminded us that pure air and sunlight were primitive man's only sure defence against disease; yet of this primary defence we have in great measure denuded ourselves by thickening the air and obscuring the sunshine. We discuss almost with passion the cubical contents of the workman's house and discuss the need of parlours as though they were of the essence of a healthy life, yet parlours will not save us from disease, while clean air will. Happily the new Housing Act will, it seems likely, shortly be reinforced by more stringent legislation for the abatement of smoke. Upon the solution of these twin problems of housing and smoke depends, in the main, the comfort and health of the next generation. "To combat bad housing is to-day as urgent a problem as it was to fight the Germans in 1914." The dictum is that of the Prime Minister, who has lost no opportunity of impressing upon the country how vast and how difficult is the problem not only of providing dwellings for those who are longing for them, but of providing them of a good type, and getting rid of the bad ones. The latest Housing Act should do something substantial towards filling the wide gap between expanding population and the lack of roof-trees, but in the nature of things it will be only a beginning, and it is to private enterprise rather than to municipal schemes that we must look for real help. Since the beginning of last October some 15,000 houses have been built by private capital, and 18,000 more are in course of erection. The encouragement which the new Act will give to the builder should, during the next twelve months, make a highly gratifying addition to these figures. Nor must we forget that more than 21,000 municipal houses have already been authorised under the measure which has recently received the Royal Assent.

But, however hard we may work at housing, whatever encouragements we may give to the builder and the investor to erect new houses, we shall still be faced with the grave difficulty of the slums. The mere making up of the arrears of building will not solve that problem. At the most moderate calculation six or seven years will be needed for a return to normality, since we have not only to wipe out arrears but to keep abreast of the current demand —a demand which grows larger every year by reason of the proportionate increase of the population. The slum is likely to be the outstanding social and sanitary crux of the near future. Nothing stands more obstinately in the way of the reduction of disease and the lowering of the death-rate than the continued existence, in almost every town of more than a few thousand inhabitants, and often in much smaller ones, of wretched dwellings, airless and decayed, nearly all of which are overcrowded. For several years past the law has been almost powerless to deal with these herding places of disease in which children grow up rickety and anæmic, with grossly insufficient breathing-space and little access to sunlight. People must have a roof over their heads, even if it leaks, and it is impossible to close and demolish these rookeries until we can replace them with something more sanitary and more Christian. A row of wooden huts in a field would be infinitely preferable to dwellings in a fetid court, especially in hot weather. For the moment, therefore, we have to be patient and do the best we can in circumstances so unhappy that we are all conscious that they cannot continue much longer. The time has come when statesmen—if there are any left—ought to be prepared to tackle the problem with serious determination to make an end of it. The elimination of slums is a national as well as a local necessity, and a really large scheme would probably involve a comparatively small loss in the end. Most slums are in central positions in which re-planning would produce an amount of technical "Betterment" which would go far towards redeeming the cost. But, however that might work out, it is certain that we are spending more upon hospitals, asylums, and prisons than would be needed to clear a vast number of slums, putting aside altogether the incalculable loss we are suffering in the weakening of the national fibre and the growth of social discontent. For the sake of a relatively few millions to-day we are laying up for ourselves a future in which the only element that is not doubtful is the continuance, and perhaps the increase, of a vast mass of avoidable disease and preventible crime.

